# MRI Evaluation and Characterization of Ovarian Lesions Based on Ovarian-Adnexal Reporting and Data System MRI

**DOI:** 10.7759/cureus.67904

**Published:** 2024-08-27

**Authors:** Purnachandra Lamghare, Sayali Paidlewar, Rahul Arkar, Varsha Rangankar, Ojasvi Sharma, Sravya Julakanti, Ankita Pandey

**Affiliations:** 1 Radiology, Dr. D. Y. Patil Medical College, Hospital and Research Centre, Dr. D. Y. Patil Vidyapeeth (Deemed to Be University), Pune, IND

**Keywords:** malignant ovarian lesions, adnexal masses, o-rads, mri, ovarian lesions

## Abstract

Background

Managing ovarian lesions requires differentiating between benign and malignant cases. The development of a multiparametric MRI approach combining anatomical and functional criteria has led to the creation of the Ovarian-Adnexal Reporting and Data System (O-RADS) MRI scoring system, which enhances diagnostic accuracy.

Objectives

To study ovarian lesions and their characteristics, along with their risk stratification based on MRI O-RADS.

Methods

A prospective study used the O-RADS MRI criteria to categorize ovarian lesions. Clinical findings and MRI results were compared with histopathological outcomes to assess diagnostic accuracy.

Results

We identified abdominal pain as the most prevalent clinical finding among our cases (64, 91.43%), followed by a lump in the abdomen (33, 47.5%), dysmenorrhea (33, 47.5%), bleeding per vaginal (15, 21.43%), and weight loss (11, 15.71%). A total of 80 ovarian lesions were examined and characterized on the basis of the O-RADS MRI risk stratification system. Among the 80 ovarian lesions, 54 were histopathologically confirmed ovarian lesions (39 (72.22%) were benign, and 15 (27.77%) were malignant). The most common benign lesions were ovarian serous cystadenoma (28.20%) and ovarian mucinous cystadenoma (20.51%), while the most common malignant lesions were serous carcinoma (33.33%) and mucinous carcinoma (20%). Using the O-RADS MRI scoring system, we categorized six lesions (7.5%) as O-RADS 1 (all benign), 34 lesions (42.50%) as O-RADS 2 (32 benign and 2 malignant), 24 lesions (30%) as O-RADS 3 (23 benign and 1 malignant), seven lesions (8.75%) as O-RADS 4 (four benign and three malignant), and nine lesions (11.25%) as O-RADS 5 (all malignant). Our findings revealed significant differences in the size of lesions, the presence of thick septa, high T2-weighted signal intensity within solid tissue, and patterns of solid component enhancement and wall irregularity between malignant and benign lesions. The MRI cut-off score of ≥4 for malignancy demonstrated a sensitivity of 94.59%, a specificity of 97.5%, an accuracy of 97.62%, a positive predictive value of 94.5%, and a negative predictive value of 97.5%. The positive likelihood ratio was 32.7, while the negative likelihood ratio was 0.025. These results affirm the high diagnostic accuracy of the O-RADS MRI scoring system in distinguishing benign from malignant ovarian lesions.

Conclusion

The O-RADS MRI score is a highly accurate tool for differentiating between benign and malignant ovarian lesions. Its application can significantly enhance the management and treatment outcomes for patients with adnexal masses. The study confirms the scoring system's high sensitivity, specificity, and overall diagnostic accuracy.

## Introduction

The ovary contains a variety of cell types with the potential to develop into tumors due to the ongoing, cyclical changes in ovarian function from adolescence to menopause. Ovarian tumors rank as the sixth most common cause of cancer-related deaths in women worldwide, with a lifetime risk of 1 in 75 for developing an ovarian tumor and 1 in 100 for dying from it [1]. In India, ovarian cancer is particularly concerning, with 103,716 cases reported in 2020, including 45,701 new cases. The country has a mortality rate of approximately 32,077 deaths from ovarian tumors, translating to 15.65 cases per 100,000 females. According to the 2018 World Ovarian Cancer Coalition Atlas, India has the second-highest incidence of ovarian cancer globally, with Pune and Delhi reporting the highest frequencies [2].

In Western countries, approximately 10% of women undergo surgery for adnexal masses, but ovarian cancer accounts for less than 15% of these cases, with a cancer incidence of only 3.6% in cystic tumors post-surgery [3]. Despite these statistics, ovarian cancer remains the leading cause of death from gynecological cancers in affluent nations [4]. The challenge in managing adnexal masses lies in distinguishing benign lesions from malignant ones. Misdiagnosing benign lesions as malignant can lead to overtreatment while mistaking malignant lesions for benign can result in inadequate treatment and poor outcomes [5].

To address these challenges, non-invasive diagnostic methods have gained importance. An accurate diagnosis before initiating surgical intervention is crucial. Serum CA125 levels, commonly used for ovarian cancer diagnosis, are not always reliable, particularly in early-stage cancers, and can also be elevated in benign gynecological conditions [6]. Ultrasonography (USG) is typically the first test for assessing adnexal masses, as it effectively classifies most masses as benign or malignant. Pattern recognition in the US, performed by skilled ultrasonographers, has been identified as a reliable technique for differentiating lesions [7]. However, variations in expertise among sonographers highlight the need for standardized risk-evaluation algorithms to improve classification accuracy [8].

The Risk of Malignancy Index (RMI), introduced in 1990, provided a tool for assessing the risk of malignancy but lacked reliability [9]. With advancements in imaging technology, MRI has become invaluable for resolving indeterminate ultrasound findings, distinguishing solid masses, and optimizing patient care [10]. MRI's ability to provide precise diagnoses for ambiguous lesions reduces unnecessary procedures for benign conditions. Key to MRI-based risk classification is the presence of enhancing solid tissue; lesions without solid tissue have a near-zero malignancy risk. MRI excels in defining abnormalities in various female reproductive system issues, including ovarian mass lesions, myomas, and endometrial cancer [10].

The ADNEX MR system, a standardized tool for assessing ovarian lesions, and the Ovarian-Adnexal Reporting and Data System (O-RADS) MRI stratification system, introduced a year later, further enhanced diagnostic accuracy [11,12]. Standardized ratings facilitate accurate malignancy risk classification, even for less experienced radiologists, and ensure consistency in interpretation. A thorough understanding of typical imaging findings and the pathological features of ovarian neoplasms, including metastases, aids in narrowing the differential diagnosis and determining appropriate surgical management [13]. Precise preoperative diagnosis influences whether surgery is performed by a surgical oncologist, gynecologic oncologist, or gynecologist, and may also identify related synchronous cancers, impacting treatment outcomes.

## Materials and methods

This prospective study was conducted at Dr. D. Y. Patil Medical College and Hospital and Research Centre, Pune, India, from August 2022 to July 2024. Siemens MAGNETOM Vida Magnetic Resonance Imaging (3 Tesla) Scanner (Siemens Healthineers, Erlangen, Germany) was used as the primary method of diagnosis. The study was granted ethical clearance by the Institutional Ethics Committee of Dr. D. Y. Patil Medical College, Hospital and Research Centre with research protocol number IESC/PGS/2022/171. Informed and written consent was sought from all patients or their guardians before they participated in the study.

Participation criteria

Females who were clinically diagnosed with ovarian pathologies and patients with a provisional diagnosis of ovarian lesions on ultrasonography of the abdomen/pelvis were included. Exclusion criteria included patients with a history of cardiac pacemakers, metallic foreign bodies, non-compatible dental implants, orthopedic hardware, claustrophobia, congenital paramesonephric gonadal ridge anomalies, chronic renal failure, hemodynamically unstable patients, and those with a known allergic reaction to dye. The final population consisted of 70 women with 80 ovarian lesions (10 women had bilateral ovarian involvement). Among the 80 lesions, the diagnosis was finalized based on imaging follow-up findings (≥1 year) in 26 lesions (32.5%) and histopathological findings in 54 lesions (67.5%).

Sample size

The present prospective study observed 101 women who underwent a pelvic MRI (plain + contrast). We excluded 16 women who did not undergo any follow-up imaging at our center within 6 months and 15 women due to lack of surgical or imaging follow-up. Thus, the final population consisted of 70 women with 80 ovarian lesions (10 women had bilateral ovarian involvement). Amongst the 80 lesions, diagnosis was finalized based on Imaging follow-up findings (≥1 year) in 26 lesions (32.5%) and histopathological findings in 54 lesions (67.5%).

To determine the sample size (n) for this study, we considered several factors. Given the expected prevalence of malignant ovarian lesions at around 20% (p) [14], with a 95% confidence level (Z = 1.96). We selected a margin of error of 10% (E) to ensure sufficient precision in our results. We calculated the required sample size to be approximately 62. Adjusting for potential dropouts, we rounded this up to 70 to ensure robust and reliable findings. The formula used was $n = \frac{Z^2 \times p \times (1 - p)}{E^2}$.

Data collection

For the MRI pelvis scan technique, participants were positioned in a supine manner with their heads oriented toward the magnet to ensure minimal movement during the examination. Utilization of body coils on the abdominal and pelvic regions was accompanied by securing them with straps to minimize any artifacts caused by respiration. The central location of the coil was specifically set at a distance of 10 cm below the level of the iliac crest. Imaging procedures were conducted using a 3T scanner equipped with a pelvic phased-array coil. A fasting period of 4-6 hours was required before the MRI scans. There was no administration of antispasmodic medications, vaginal contrast, or rectal contrast. The imaging protocol was designed to evaluate adnexal masses through a series of sequences including T2-weighted scans in axial, sagittal, and coronal planes, in-phase and out-of-phase T1-weighted sequences, diffusion-weighted sequences with varying b values (0, 500, 400, and 800 s/mm²), and T1-weighted sequences both with and without fat saturation, both pre and post intravenous injection of Gadopentetate dimeglumine (0.1 mL/kg MulitHance) delivered at a rate of 3 mL/sec per kg body weight. Images of the representative cases are shown in the Appendix.

Statistical analysis

The analysis of data was conducted using IBM SPSS Statistics for Windows, Version 25 (Released 2017; IBM Corp., Armonk, New York, United States). Continuous variables were represented by means ± standard deviations, whereas categorical variables were exhibited in frequencies and percentages.

Specificity, sensitivity, positive predictive value, negative predictive value, and overall accuracy were calculated to assess the diagnostic accuracy of the O-RADS MRI scoring system. MRI scoring system was evaluated by utilizing receiver operating characteristics. The optimal cut-off point for distinguishing between benign and malignant lesions was determined by the area under the curve (AUC).

The chi-square test and t-test were used to calculate the statistical significance. A p-value less than 0.05 was considered to be statistically significant.

## Results

The mean age of our patients was 36.85 ± 15.45 years. The majority were within the 25-34 years age group (32, 45.07%), followed by those in the 35-44 years age group (11, 15.49%). The youngest patient was 17 years old, and the oldest was 84 years old. Clinical findings reveal that abdominal distension was present in 30 (37.5%) patients, while 50 (62.5%) did not experience this symptom. Dysmenorrhea was reported by 33 (47.14%) patients, with the remaining 37 (52.85%) not having this condition. A lump in the abdomen was found in 47.5% (33) of the cases, whereas 37 (52.5%) did not have this finding. Pain in the abdomen was the most common symptom, affecting 64 (91.43%) patients, leaving only 6 (8.57%) without this complaint. Bleeding per vaginal (PV), not related to normal menstrual cycle or any other hormonal issues, was noted in 15 (21.43%) of the patients, while 55 (8.57%) did not report this symptom. Last, weight loss was observed in 11 (15.71%) patients, with 59 (84.29%) not experiencing this issue (Table 1).

**Table 1:** Distribution of clinical findings

Clinical findings	Present	Absent
Abdominal distension	26 (37.5%)	44 (62.5%)
Dysmenorrhea	33 (47.14%)	37 (52.85%)
Lump in abdomen	33 (47.5%)	37 (52.5%)
Pain in abdomen	64 (91.43%)	6 (8.57%)
Bleeding per vaginal	15 (21.43%)	55 (78.57%)
Weight loss	11 (15.71%)	59 (84.29%)

The characterization of ovarian lesions on the basis of the O-RADS MRI risk stratification system [7], reveals six cases categorized as O-RADS MR 1, all of them were benign, with no instances of malignancy reported. Specifically, there were two cases (33.33%) of ovarian follicles, which are described as simple cysts measuring 3 cm in premenopausal women. Additionally, there were two cases (33.33%) of corpus luteum and two cases (33.33%) of hemorrhagic cysts less than 3 cm in premenopausal women (Table 2).

**Table 2:** Ovarian lesion characterized as O-RADS 1 O-RADS: Ovarian-Adnexal Reporting and Data System

MRI presentation	Benign (%)	Malignant
O-RADS MR 1 (n = 6)	6 (100%)	0
Ovarian follicle (simple cyst 3 cm in premenopausal women)	2 (33.33%)	0
Corpus luteum	2 (33.33%)	0
Hemorrhagic cyst <3 cm in premenopausal women	2 (33.33%)	0

The MRI presentation data reveals the distribution of benign and malignant findings within the O-RADS MR 2 category, which includes a total of 34 cases. Of these cases, 32 (94.11%) were identified as benign, while two (5.89%) were malignant. The benign cases are further categorized into various types: simple unilocular cysts constituted nine (26.47%) cases; para-tubal or para-ovarian cysts made up six (17.64%); hemorrhagic cysts larger than 3 cm represented three (8.82%) cases; typical endometriomas accounted for seven (20.58%) cases; and typical mature dermoid cysts accounted for four (11.76%) cases. Additionally, dilated fallopian tubes with simple fluid content were seen in three (8.82%) cases. The only malignant cases observed were lesions exhibiting "dark T2/dark DWI" solid tissue, which comprised two (5.88%) cases of all. No malignant cases were found among the other categories of benign lesions (Table 3).

**Table 3:** Ovarian lesion characterized ss O-RADS 2 O-RADS: Ovarian-Adnexal Reporting and Data System

MRI presentation	Benign (%)	Malignant (%)
O-RADS MR 2 (n = 34)	32 (94.11%)	2 (5.89%)
Simple unilocular cyst (any type of fluid content, no wall enhancement)	9 (26.47%)	0
Para-tubal or para-ovarian cyst	6 (17.64%)	0
Hemorrhagic cyst >3 cm	3 (8.82%)	0
Typical endometrioma	7 (20.58%)	0
Typical mature dermoid cyst	4 (11.76%)	0
Dilated fallopian tubes with simple fluid content	3 (8.82%)	0
Lesion with "dark T2/dark DWI" solid tissue	0	2 (5.88%)

In the O-RADS 3 category, which includes a total of 24 lesions, 23 (95.83%) were benign, and one (4.17%) was malignant. Among the benign lesions, 11 (45.83%) were classified as multilocular cysts without any solid tissue. In contrast, the single malignant lesion (4.17%) was identified as a multilocular cyst with non-enhancing solid components. Additionally, two lesions (8.33%) exhibited features of dilated fallopian tubes with non-simple fluid content. This distribution highlights that most lesions in the O-RADS 3 category are benign, with only a small proportion being malignant, often associated with more complex features (Table 4).

**Table 4:** Ovarian lesion characterized as O-RADS 3 O-RADS: Ovarian-Adnexal Reporting and Data System

MRI presentation	Benign n (%)	Malignant n (%)
O-RADS MR 3 (n = 24)	23 (95.83%)	1 (4.17%)
Multilocular cysts without solid tissue	11 (45.83%)	1 (4.17%)
Non-enhancing solid tissue	1 (4.17%)	0
Unilocular cyst (any type of fluid content (mon-simple) with smooth enhancing wall or non-typical of endometrioma or dermoid cyst	9 (37.5%)	0
Dilated fallopian tube with a non-simple fluid content	2 (8.33%)	0

In this analysis of MRI presentations under the O-RADS MR 4 category, a total of seven cases were evaluated. The overall classification revealed that 57.14% (four out of seven) of the cases were benign, while 42.85% (three out of seven) were malignant. When focusing specifically on solid tissue (excluding dark T2/dark DWI) that enhances the level of the myometrium at 30-40 seconds on non-dynamic contrast-enhanced (non-DCE) imaging, the distribution remained consistent with four cases (57.14%) being benign and three cases (42.85%) being malignant. There were no cases identified with large volume enhancing solid tissue in a lesion containing lipid content (Table 5).

**Table 5:** Ovarian lesion characterized as O-RADS 4 O-RADS: Ovarian-Adnexal Reporting and Data System; DCE: dynamic contrast-enhanced; DWI: diffusion-weighted imaging

MRI presentation	Benign (%)	Malignant (%)
O-RADS MR 4 (n = 7)	4 (57.14%)	3 (42.85%)
Solid tissue (excluding dark T2/dark DWI) enhancing ≤ myometrium at 30-40 sec on non DCE	4 (57.14%)	3 (42.85%)
Large volume enhancing solid tissue in a lesion with lipid content	0	0

All nine cases (n = 9) classified as O-RADS MR 5 were found to be malignant, with no benign cases observed. This category specifically includes solid tissue (excluding dark T2/dark DWI) that exhibits high-risk time-intensity curves (HR TIC) on DCE MRI or enhancement greater than the myometrium at 30-40 seconds on non-DCE imaging (Table 6).

**Table 6:** Ovarian lesion characterized as O-RADS 5 O-RADS: Ovarian-Adnexal Reporting and Data System; DCE: dynamic contrast-enhanced; DWI: diffusion-weighted imaging; HR TIC: high-risk time-intensity curves

MRI presentation	Benign (%)	Malignant (%)
O-RADS MR 5 (n = 9)	0 (0%)	9 (100%)
Solid tissue (excluding dark T2/dark DWI) with HR TIC on DCE or enhancing > myometrium at 30-40 sec on non-DCE	0 (0%)	9 (100%)

The final diagnosis of the ovarian lesions was determined through two primary methods: histopathological examination for 54 lesions (67.5%) and clinical imaging follow-up for 26 lesions (32.5%). Among the 54 lesions confirmed by histopathology, 39 (72.22%) were benign and 15 (27.77%) were malignant. Among the 39 benign lesions, 34 (87.17%) were of ovarian origin, while five (12.82%) were non-ovarian. For the ovarian benign lesions, the most common types were serous cystadenomas, accounting for 11 cases (28.20%), followed by mucinous cystadenomas with eight cases (20.51%). Additionally, there were two cases each (5.12%) of serous cystadenofibroma, functional cysts, and ovarian torsion. Endometriomas and hemorrhagic cysts were each present in four cases (10.25%). A single case (2.56%) was identified as a germ cell tumor. The non-ovarian benign lesions included three cases of hydrosalpinx (7.69%) and two cases of hemato-salpinx (5.12%).

In contrast, the 15 malignant lesions showed a different distribution. Of these, 13 lesions (86.66%) were ovarian, while two lesions (13.33%) were non-ovarian. Among the malignant ovarian lesions, serous carcinomas were the most prevalent, accounting for five cases (33.33%). Mucinous carcinomas were the next most common, with three cases (20%). There was also one case each of carcinosarcoma and clear cell carcinoma (6.66% each). Additionally, one case (6.66%) was identified as a malignant germ cell tumor, and two cases (13.33%) were classified as metastases. The non-ovarian malignant lesions comprised two cases of tubal cancer (13.33%) (Table 7).

**Table 7:** Final diagnosis for ovarian lesions based on histopathology

Final diagnosis	n (%)
Method of establishing diagnosis	
Imaging follow-up findings (≥1 year)	26 (32.5%)
Histopathologic results	54 (67.5%)
Benign disease (n = 39)	
Ovarian serous cystadenoma	11 (28.20%)
Ovarian mucinous cystadenoma	8 (20.51%)
Serous cystadenofibroma	2 (5.12%)
Germ cell tumor	1 (2.56%)
Endometrioma	4 (10.25%)
Hemorrhagic cyst	4 (10.25%)
Functional	2 (5.12%)
Ovarian torsion	2 (5.12%)
Non-ovarian benign	
Hydrosalpinx	3 (7.69%)
Hematosalpinx	2 (5.12%)
Malignant disease (n = 15)	
Ovarian	
Serous	5 (33.33%)
Mucinous	3 (20.00%)
Carcinosarcoma	1 (6.66%)
Clear cell	1 (6.66%)
Germ cell tumor	1 (6.66%)
Metastasis	2 (13.33%)
Non-ovarian malignant	
Tubal cancer	2 (13.33%)

The characterization of ovarian lesions based on LEXICON parameters [7] revealed several distinct features that differentiate malignant from benign lesions. The size of malignant lesions (93.25 ± 31 mm) was significantly larger compared to benign lesions (70 ± 23.50 mm), with a p-value of 0.034. The presence of a septum and its thickness were also distinguishing factors: 12 (80%) malignant cases had a septum compared to 16 (24.61%) benign cases, and thick septum was more common in malignant lesions (9, 75%) than in benign ones (3, 18.75%), both with a p-value of 0.001. The T2-weighted signal intensity within solid tissue did not significantly differ between benign (12, 15.38% low and 55, 84.61% medium/high) and malignant cases (2, 13.33% low and 13, 86.66% medium/high), with a p-value of 0.541.

Solid component enhancement patterns showed contrasting observations. For those enhancing less than or equal to myometrium at 30-40 seconds on non-DCE MRI, four (57.14%) were benign and three (42.86%) were malignant (p = 0.001). Notably, all cases enhancing greater than myometrium at 30-40 seconds were malignant (100%), with none benign. Wall characteristics further differentiated the groups; wall enhancement was present in 100% of malignant cases compared to 27 (41.53%) benign cases (p = 0.0023), and wall irregularity was observed in 13 (86.66%) malignant cases versus 5 (7.70%) benign cases (p = 0.001). Ascites were more common in malignant cases (11, 73.33%) compared to benign cases (28, 43.07%), although this was not statistically significant (p = 0.489). Finally, peritoneal implants and metastasis were exclusively found in malignant cases (100%), with both parameters showing a p-value of 0.001. These findings underscore the critical role of MRI features in distinguishing between benign and malignant diseases, aiding in more accurate diagnosis and management (Table 8).

**Table 8:** MRI characteristics of ovarian lesions ^*^ indicates statistically significant DCE: dynamic contrast-enhanced

MRI parameters	Benign disease (n = 65)	Malignant disease (n = 15)	p-value
Size (mm)	70 ± 23.50	93.25 ± 31	0.034^*^
Septum			
Absent	49 (75.38%)	3 (20%)	0.001^*^
Present	16 (24.61%)	12 (80%)	
Septum thickness			
Thin	13 (81.25%)	3 (25%)	0.001^*^
Thick	3 (18.75%)	9 (75%)	
T2-weighted signal intensity within solid tissue			
Low	10 (15.38%)	2 (13.33%)	0.541
Medium/high	55 (84.61%)	13 (86.66%)	
Solid component enhancement pattern (excluding T2/DWI dark)			
Enhancing ≤ myometrium at 30-40s on non-DCE MRI (n = 7)	4 (57.14%)	3 (42.86%)	0.001^*^
Enhancing ≥ myometrium at 30-40s on non-DCE MRI (n = 9)	0%	9 (100%)	
Wall characteristics			
Wall enhancement	-		
Yes	27 (41.53%)	15 (100%)	0.0023^*^
No	38 (58.46%)	0%	
Wall irregularity	-		
Yes	5 (7.70%)	13 (86.66%)	0.001^*^
No	60 (92.30%)	2 (13.33%)	
Ascites			
Yes	28 (43.07%)	11 (73.33%)	0.489
No	37 (56.93%)	4 (26.67%)	
Peritoneal implants (n = 5)	0%	100%	0.001^*^
Metastasis (n = 10)	0%	100%	0.001^*^

The MRI stratification of ovarian lesions based on the O-RADS scoring system provided valuable insight into the malignancy risk associated with different O-RADS categories. Evaluating 80 ovarian lesions, 19 (23.75%) were malignant, while 61 (76.25%) were benign. The lesions were classified into O-RADS categories to assess their malignancy risk and determine the appropriate diagnostic approach.

**Figure 1:**
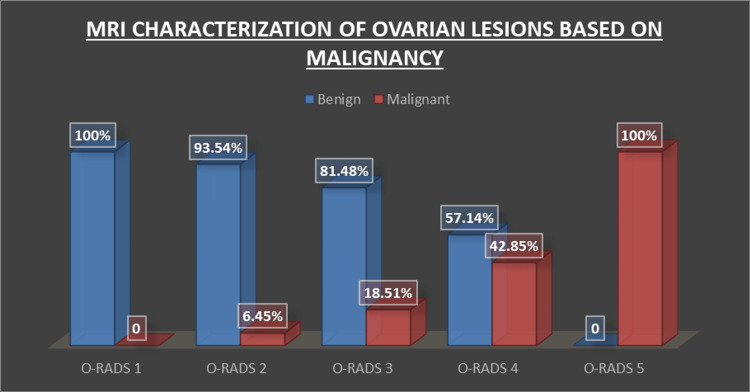
MRI characterization of ovarian lesions based on malignancy O-RADS: Ovarian-Adnexal Reporting and Data System

O-RADS 1 included six lesions (7.5%), all of which were benign. There were no malignant lesions in this group, indicating that lesions classified as O-RADS 1 are considered very low risk for malignancy. O-RADS 2 comprised 31 lesions (38.75%), with two lesions (6.46%) found to be malignant and 29 lesions (93.54%) benign. O-RADS 3 included 27 lesions (33.75%), with five lesions (14.81%) being malignant and 22 lesions (81.48%) benign. O-RADS 4 had seven lesions (8.75%), with three lesions (42.85%) found to be malignant and four lesions (57.14%) benign. O-RADS 5 included nine lesions (11.25%), all of which were malignant (100%) (Figure 1).

**Figure 2:**
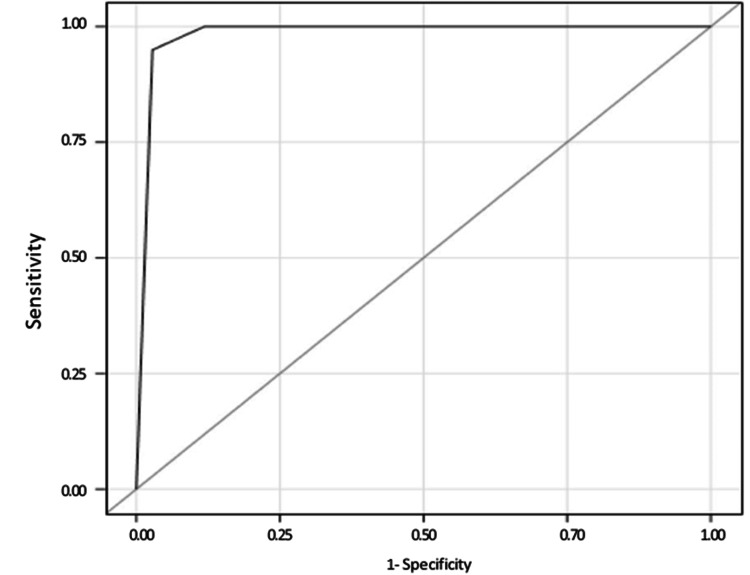
ROC curve ROC: receiver operating characteristic

The diagnosis of functional ovarian lesions (n = 54) was confirmed through surgical intervention, histopathology, or clinical follow-up, while a one-year negative follow-up using MRI confirmed diagnoses in another set (n = 26). For identifying malignant conditions, an MRI cut-off score of ≥4 demonstrated 94.59% sensitivity (95% CI, 87.54%-99.200%), 97.5% specificity (95% CI, 93.65%-99.31%), and 97.62% accuracy (95% CI, 93.46%-98.53%). The positive predictive value was 94.5%, and the negative predictive value was 97.5%. The positive likelihood ratio was 32.7 (95% CI, 14.23-48.81), and the negative likelihood ratio was 0.025 (95% CI, 0.02-0.35). Two malignant ovarian tumors with an O-RADS 2 score were identified as serous adenocarcinoma of the bilateral ovaries and fallopian tubes. A high-grade in-situ mucinous adenocarcinoma with occasional foci of micro invasion received an O-RADS 3 score due to its multilocular cyst nature, high mucinous content, and multiple enhancing septae post-contrast. Four benign lesions scored as O-RADS 4 including three mucinous cystadenomas and one serous cystadenoma. The ROC curve analysis of MRI O-RADS scores yielded an area of 0.974 (97% CI, 0.96-0.99), highlighting a cutoff value of ≥4 as the most effective threshold for identifying malignancy (Table 9, Figure 2).

**Table 9:** Diagnostic performance of O-RADS MRI PPV: positive predictive values; NPV: negative predictive values; O-RADS: Ovarian-Adnexal Reporting and Data System; AUC: area under the curve

Cut point	Sensitivity (%)	Specificity (%)	PPV (%)	NPV (%)	Youden's Index	AUC	Metric score
≥4	94.59	97.5	94.5	97.5	0.92	0.974	0.969

## Discussion

To manage ovarian lesions, it is essential to differentiate them according to their malignancy or benignity. The exact description of ovarian lesions significantly influences the quality of patient treatment and survival outcomes. Despite being established as a reliable secondary approach for many years, MRI was not included in clinical recommendations and standardized protocols for the management of adnexal lesions. By developing a multiparametric approach combining anatomical and functional MRI criteria, a scoring system was devised that substantially enhances the accuracy of predicting the absence of cancer. This advancement establishes MRI as a highly useful tool for guiding patient therapy.

To create the O-RADS MRI score, Thomassin-Naggara et al. (2020) prospectively evaluated an upgraded version of the ovarian mass grading system utilizing a sizable multi-center patient sample. The new grading method, based on their MRI findings, demonstrated high diagnostic accuracy. Few investigations have, however, demonstrated its diagnostic value. Therefore, we aimed to use O-RADS criteria to define ovarian lesions in the current investigation [15].

In the present investigation, we found that the most prevalent clinical finding among our cases (91.43%) was non-cyclical abdominal pain, followed by a lump in the abdomen (47.5%), dysmenorrhea (35.72%), bleeding PV (21.43%), and weight loss (15.71%). In a study conducted by Ramadan et al. (2024), 145 patients underwent pelvic MRI due to symptoms of either pelvic pain or vaginal bleeding [16]. Similar to our results, Hassan et al. (2024) observed that pain was present in all 72 patients (100%), while constipation or diarrhea, fever, presence of palpable mass or enlarged abdominal volume, vaginal bleeding, and urinary symptoms were present in 19.4%, 63.9%, 44.4%, 41.7%, and 22.2% of women, respectively [17].

Among the 35 benign lesions, the most common were 11 (28.20%) ovarian serous cystadenomata, followed by eight ovarian mucinous cystadenoma lesions (20.51%). Among 15 malignant lesions, five lesions were serous (33.33%), three lesions were mucinous (20%), and carcinosarcoma and clear cell were one each (6.66%). According to Pereira et al. (2022), the most common benign tumor of epithelial origin was a germ cell tumor. The most prevalent malignant tumor in women was serous adenocarcinoma, followed by clear cell and metastatic tumor [18].

Consistent with our study, Hassan et al. (2024) classified 32 lesions (44.4%) as O-RADS 2 by MRI, and two were diagnosed as malignant by pathology, while eight benign lesions were classified as O-RADS 3 (11.1%) by MRI. In O-RADS 4, two of the six lesions (8.3%) were found to be benign by pathologists. Six lesions (36.1%) classified as O-RADS 5 by MRI were pathologically confirmed to be benign [16]. According to Hottat et al., MRI was used to examine 402 women who had adnexal tumors that were not well defined. In 27 individuals, 32 lesions received a score of 2, 88 received a score of 3, 30 received a score of 4, and 39 received a score of 5 in 31 individuals. Out of the 201 lesions, 58 (28.9%) were malignant, whereas the remaining lesions were benign [19].

We observed that malignant lesions had significant findings of increased lesion size, presence of thick septa, high T2-weighted signal intensity within solid tissue, higher lesion of solid component enhancement pattern (excluding T2/DWI dark), and enhanced and irregular wall pattern compared to benign lesions. Similar to our findings, Pereira et al. (2022) reported significant differences in these parameters for malignancy. This correlates with existing literature, which indicates that in various types of cystic and solid ovarian masses, an increase in solid components is associated with a higher likelihood of malignancy [18].

For distinguishing between benign and malignant lesions, Ramadan et al. (2024) reported significant interrater agreement for the primary O-RADS criteria, which include purely cystic nature, laterality, endometriotic lesions, locularity, low T2 and b1000 signal, enhancement degree relative to the myometrium, absence of wall enhancement, vegetations, grouped septae, and thickened regular and irregular septae. Taylor et al. (2021) have proposed that MRI can effectively predict the underlying pathology by identifying the presence of papillary projections and hypointense solid tissue on both T2-weighted MRI and high b-value DWI. This characteristic, known as "dark T2/dark DWI" in O-RADS MRI, is indicative of a benign fibrous tumor [20].

The results of our investigation indicate that using the MRI cut-off of ≥4 for malignant conditions was in concurrence with the previously published data. Ruiz et al. (2016) [12] observed one lesion with O-RADS 2 as malignant. Thomassin-Naggara et al. (2020) [15] observed two such malignant cases, and Sasaguri et al. (2019) [21] observed eight such cases under the O-RADS 2 category. Given that both lesions in our study appeared hypointense on T2WI and DWI due to the hemorrhagic content within them. The one malignant lesion that received a score of 3 was high-grade in-situ mucinous adenocarcinoma of the ovary with occasional foci of micro-invasion. The lesion was assigned O-RADS 3 as it was a multilocular cyst with high mucinous content and multiple septae which were enhanced on post-contrast.

According to Aslan and Tosun (2023) [22], the O-RADS MRI score has a high sensitivity, specificity, and accuracy rate for distinguishing between benign and malignant adnexal tumors. In a study conducted by Hassan et al., it was demonstrated that using a cutoff value of 4 or higher on the O-RADS MRI score for malignancy resulted in a sensitivity of 92.31%, specificity of 82.61%, PPV of 75, and NPV of 95 [16]. The overall accuracy was 86.11% with a 95% confidence interval ranging from 70.50% to 95.33%. The researchers discovered a direct correlation between the O-RADS MRI score and the likelihood of developing cancer. The probability ratio (PR) for a score of 2 was 0.01, for a score of 3, it was 0.27, for a score of 4, it was 4.42, and for a score of 5, it was 38.81.

This observational study was a single-center study, with a limited sample size, which may affect the broader applicability of its findings. Conducted at a single medical facility, the results may not be generalizable to other settings. Additionally, the study did not utilize dynamic contrast enhancement or analyze signal intensity curves, potentially constraining the depth and detail of imaging analysis. These limitations must be considered when interpreting the conclusions and their relevance to broader clinical practices.

## Conclusions

Our study underscores the utility of pelvic MRI, especially when interpreted using the Ovarian Adnexal Reporting and Data System (O-RADS) MRI parameters, in characterizing ovarian lesions. The results demonstrate that O-RADS MRI effectively predicts cancer rates, aligning well with forecasts for O-RADS MRI types 2 and 3, though revealing higher-than-expected malignancy rates for types 4 and 5. The application of the O-RADS MRI system enables individualized, patient-centered management of equivocal masses, potentially preventing unnecessary surgeries and considering less invasive or fertility-preserving options. This stratified approach streamlines patient care paths, ensuring timely and appropriate interventions based on lesion categorization. Despite MRI's higher cost compared to ultrasonography, its detailed lesion characterization significantly enhances preoperative assessment and clinical decision-making, emphasizing its essential role in accurately diagnosing and managing ovarian lesions.

## Figures and Tables

**Figure 3:**
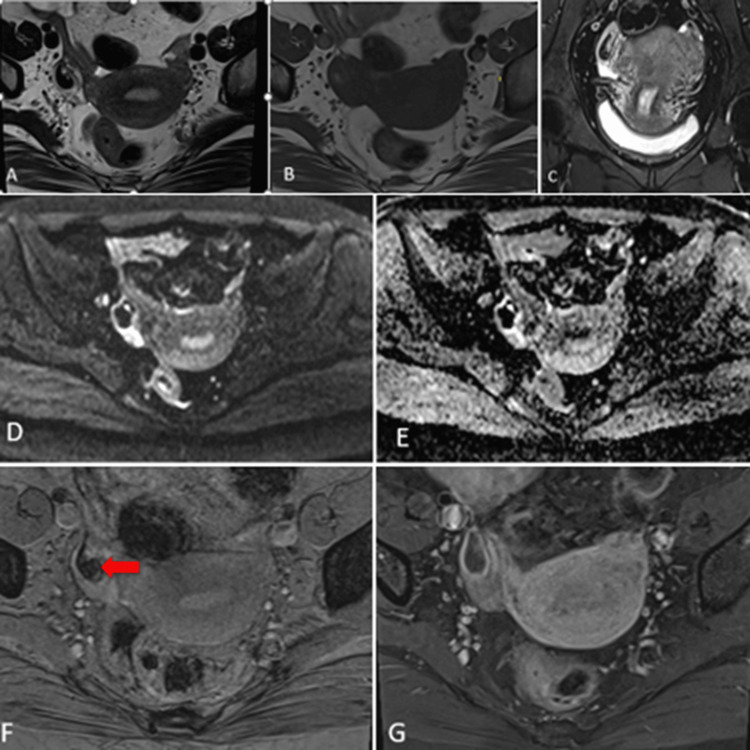
Case 1: Right hemorrhagic cyst - O-RADS 1 (benign lesion) A 32-year-old female came with secondary infertility under evaluation and presented with menstrual cycle irregularities for two months. A well-defined oval lesion of size 22 x 12 x 14 mm in the right ovary. In axial T2WI (A) it appears hyperintense, hypointense on axial T1WI (B). On coronal STIR (C) it appears hyperintense. DWI (D, E) shows no diffusion restriction. The lesion shows blooming on GRE (red arrow) (F) with subtle peripheral post-contrast enhancement on axial T1fs post-contrast (G). T2WI: T2 weighted image; STIR: short tau inversion recovery; DWI: diffusion-weighted imaging; GRE: gradient echo sequences; T1FS: T1 weighted fat-saturated image; O-RADS: Ovarian-Adnexal Reporting and Data System

**Figure 4:**
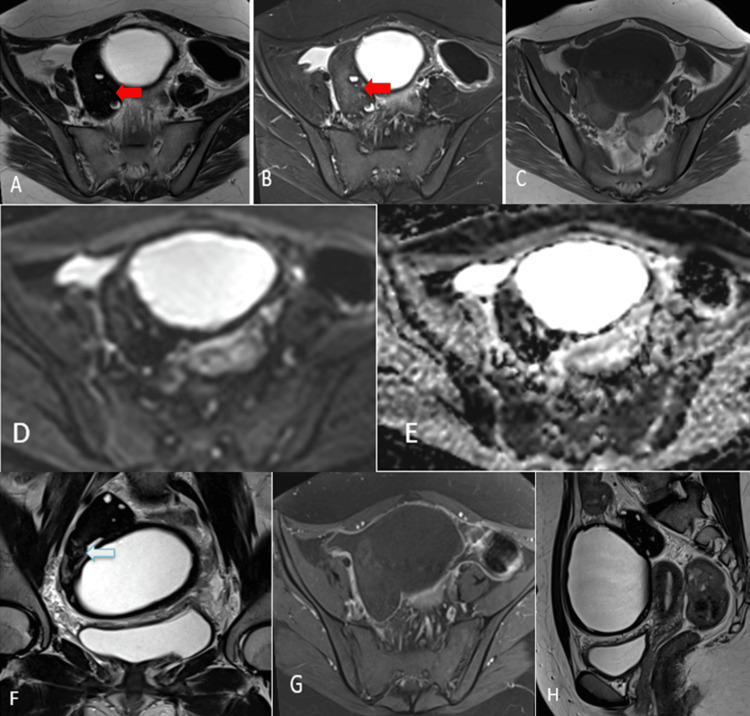
Case 2: Right para-ovarian cyst with right ovarian torsion - O-RADS 2 (benign lesion) An 18-year-old female presented with complaints of dull aching pain in the abdomen in the hypogastric region and lower back region for two months, with sudden increased discomfort in the pelvis associated with nausea, vomiting, and fever for one day. A patient with pain in the hypogastric and back region shows a well-defined oval cystic lesion in adnexa in the midline in close contiguity with the right ovary. It is seen crossing the midline and displacing the right ovary superolaterally. In axial T2WI, axial STIR (A, B) appears hyperintense. Axial T1WI (C) appears hypointense. DWI (D, E) shows no diffusion restriction. Coronal STIR (F) thickened, twisted, and bulky pedicle (blue arrow) of the right ovary. Axial T1fs post-contrast (G), the lesion shows peripheral enhancement. Sagittal T2WI (H) shows the extension of the lesion superior to the urinary bladder and anterior to the uterus. Axial T2WI, axial STIR, and axial T1WI; the right ovary appears enlarged with a significant proliferation of ovarian stroma which shows markedly low signal and peripherally displaced follicles (red arrow, A, B). Axial T1fs post-contrast (G) shows a thin rim of peripheral enhancement with no enhancement of ovarian stroma. T2WI: T2 weighted image; STIR: short tau inversion recovery; DWI: diffusion-weighted imaging; T1FS: T1 weighted fat-saturated image; O-RADS: Ovarian-Adnexal Reporting and Data System

**Figure 5:**
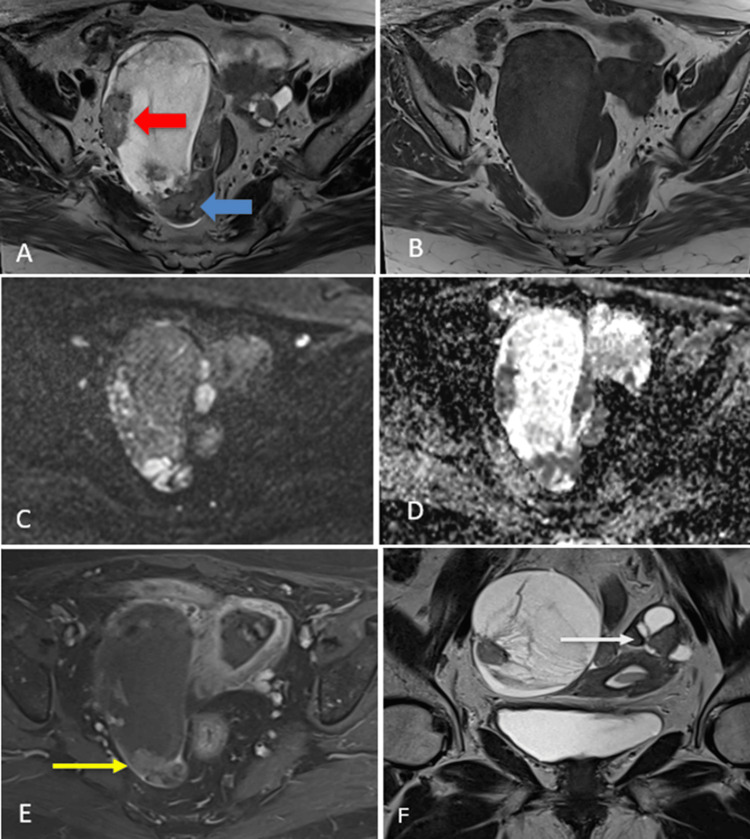
Case 3: Bilateral serous adenocarcinoma of fallopian tube and ovaries - O-RADS - 2 (malignant lesion) A 72-year-old female presented with dull aching lower abdomen pain for two years which was insidious in onset and increased gradually and was associated with a few episodes of bleeding per vaginal and distension of the abdomen. The patient also had a history of significant weight loss. A fairly well-defined round-to-ovoid tubular solid cystic mass lesion is in the right adnexa. The cystic component of the lesion appears heterogeneously hyperintense on axial T2W images (A) and hypointense on axial T1W images (B). A solid component is noted within the lesion (red arrow, A), and the right fallopian tube shows diffuse mural thickening (blue arrow, A) in its entire extent. The solid component appears iso-hypointense on T2/T1 weighted images (A, B). The solid component and the thickened fallopian tube show diffusion restriction with correspondingly low ADC values (C, D). On post-contrast T1W images, the solid component shows heterogeneous enhancement (yellow arrow, E). The left adnexa shows a similar, smaller mass lesion on coronal T2WI (white arrow, F) with the same imaging features on all the sequences. Both ovaries are not seen separately from the lesions. T2WI: T2 weighted image; T1WI: T1 weighted image; O-RADS: Ovarian-Adnexal Reporting and Data System

**Figure 6:**
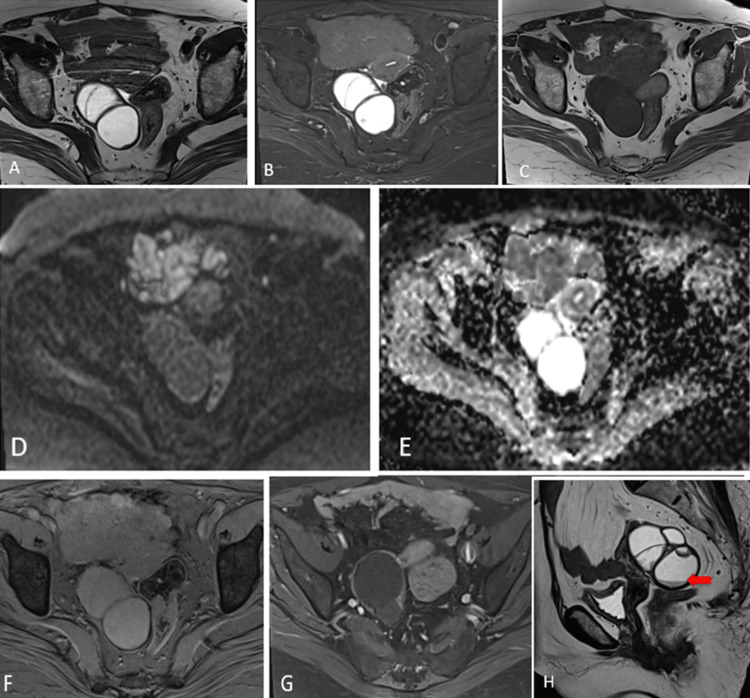
Case 4: Right serous cystadenoma - O-RADS 3 (benign lesion) A 62-year-old female presented with lower back pain radiating to the bilateral lower limb for 15 days. A large, lobulated, multi-septated lesion in the right adnexa. The lesion is in the right adnexa on axial T2WI (A) and axial STIR (B); it appears hyperintense, and on axial T1WI (C), it appears hypointense. DWI (D, E) shows no diffusion restriction. The lesion shows blooming on GRE (F) with subtle post-contrast enhancement of its thin wall on axial T1fs post-contrast (G). The septae appear thin and show no post-contrast enhancement. Sagittal T2WI (H) shows fluid-fluid level within (red arrow). T2WI: T2 weighted image; STIR: short tau inversion recovery; DWI: diffusion-weighted imaging; GRE: gradient echo sequences; O-RADS: Ovarian-Adnexal Reporting and Data System

**Figure 7:**
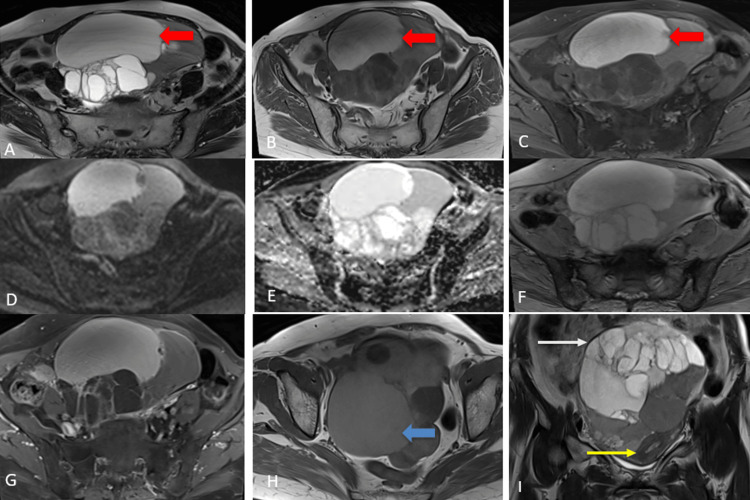
Case 5: High-grade mucinous adenocarcinoma of ovary - O-RADS 3 (malignant lesion) A 72-year-old female presented with dull aching lower abdomen pain for two years which was insidious in onset, increased gradually, and was associated with a few episodes of bleeding PV. On examination, she had a large, palpable mass in the abdomen with distension. A large, well-defined, lobulated multicystic lesion with multiple thin septae is in the pelvis superior to the uterus. On axial T2WI (A), the cysts appear hyperintense, and on axial T1WI (B), they appear hypointense. The larger cyst noted in the anterior portion of the lesion appears hyperintense on axial T2 WI, T1 WI, and axial T1 FS (red arrows A, B, and C) - suggestive of hemorrhage. No obvious diffusion restriction/blooming is noted within (D-F) Multiple thin septations show post-contrast enhancement (G). Another cyst in the posterior aspect of the lesion (blue arrow, H) appears slightly hyperintense on axial T1 WI (H) and hypointense on T2 WI (A) - suggestive of high mucin content. On coronal T2WI (I) the superior extension of the lesion can be seen until the umbilical region (white arrow, I). The uterus is compressed and displaced inferiorly and towards the left (yellow arrow, I). T2WI: T2 weighted image; STIR: short tau inversion recovery; T1FS: T1 weighted fat-saturated image; O-RADS: Ovarian-Adnexal Reporting and Data System

**Figure 8:**
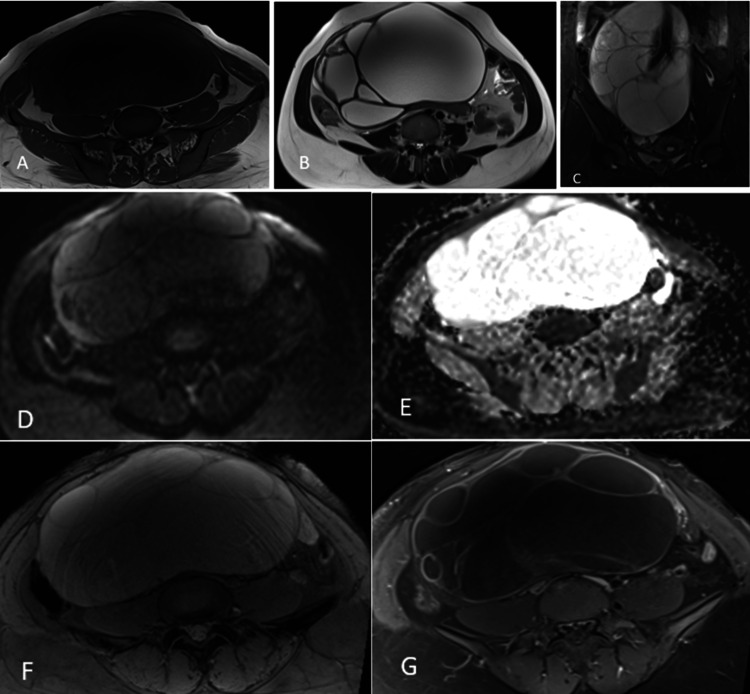
Case 6: Left mucinous cystadenoma - O-RADS 4 (benign lesion) A 48-year-old female presented with amenorrhea for six months and pain in the abdomen on and off associated with nausea for one month. A well-defined, multiloculated, multiseptated large cystic lesion in the pelvis in the midline. On axial T1WI (A), the lesion appears hypointense; on axial T2WI and STIR (B, C), it appears hyperintense. DWI (D, E) shows no diffusion restriction. The lesion shows no blooming on GRE (F). Axial T1FS post-contrast (G) shows multiple thin, enhancing septations within the lesion. The lesion is displacing the surrounding bowel loops laterally, and anteriorly, it is extending beneath the anterior abdominal wall. T2WI: T2 weighted image; STIR: short tau inversion recovery, also known as short TI inversion recovery; DWI: diffusion-weighted imaging; GRE: gradient echo sequences; O-RADS: Ovarian-Adnexal Reporting and Data System

**Figure 9:**
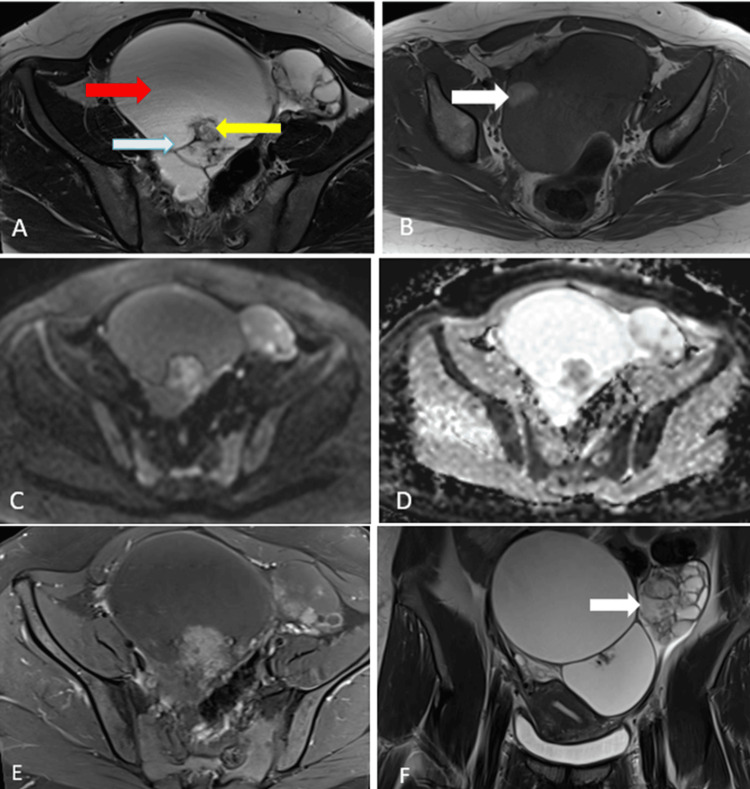
Case 7: Bilateral mucinous adenocarcinoma - O-RADS 4 (malignant lesion) A 33-year-old female presented with complaints of irregular periods for five years and hirsutism for three months. A large, well-defined, multilocular, cystic lesion in the midline of the pelvis extending on the right side is postero-superior to the uterus. On axial T2WI (A), the lesion appears hyperintense (red arrow) with multiple thin hypointense septations (blue arrow, A); on axial T1WI (B), the lesion appears hypointense. Multiple small and variable-sized solid components showing irregular and papillary projections (yellow arrow) on axial T2WI (A). DWI (C) and ADC (D) show mild diffusion restriction. Axial T1FS post-contrast (E) the solid component shows heterogenous post-contrast enhancement. A small cystic component is seen in the anterior portion of the above-mentioned multilocular cyst. It appears slightly hyperintense on T1 WI (white arrow, B), likely due to mucin. The lesion displaces the surrounding bowel loops laterally, the uterus, and anteriorly extends beneath the anterior abdominal wall. The left ovary is enlarged (white arrow) on coronal T2WI (F). It shows multiple small follicles in its periphery and a multilocular cystic lesion with thin septations and multiple solid small components within the features, which are similar to the above-mentioned lesions with the same imaging features on all the sequences. Both ovaries are not seen separately from the lesions. T2WI: T2 weighted image; DWI: diffusion-weighted imaging; T1FS: T1 weighted fat-saturated image; O-RADS: Ovarian-Adnexal Reporting and Data System

**Figure 10:**
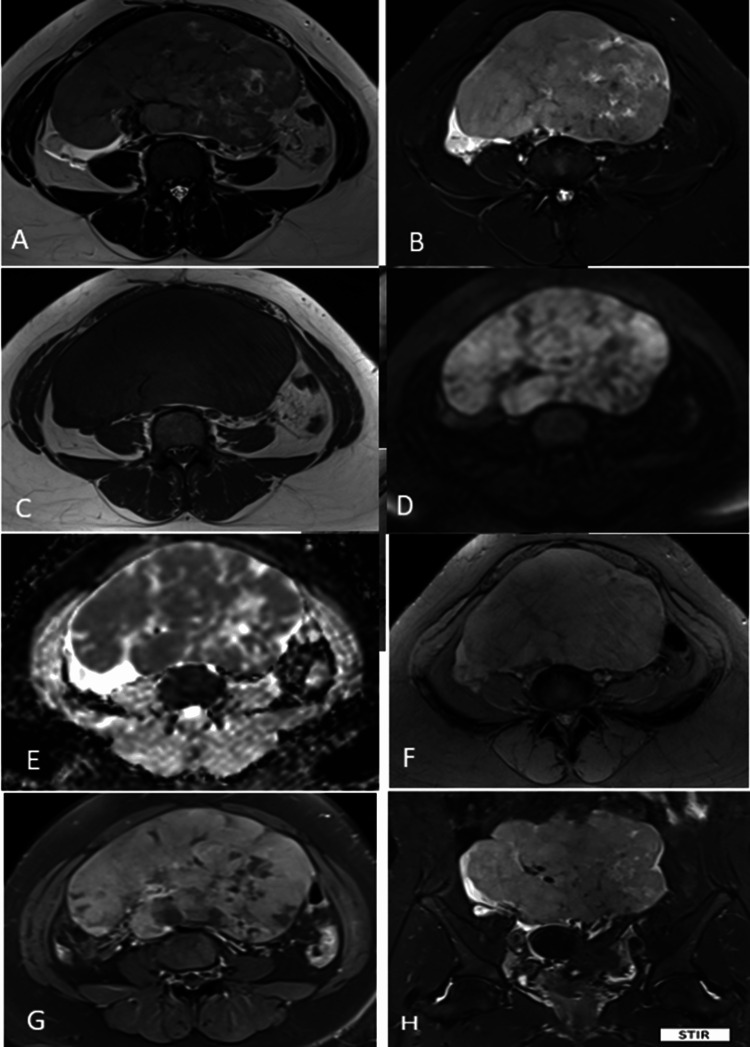
Case 8: Dysgerminoma of right ovary - O-RADS 5 (malignant lesion) A 21-year-old female with primary infertility for two years presented with complaints of pain in the lower abdomen associated with menstrual cycle irregularities and distension of the abdomen for two months. It shows a large, well-defined abdomino-pelvic mass lesion in the midline with a lobulated contour. In axial T2WI and axial STIR (A, B), the lesion appears heterogeneously hyperintense. Axial T1WI (C) appears hypo- to isointense. DWI and ADC (D, E) show no restricted diffusion. On axial GRE (F), the lesion shows the small interspersed focus of signal dropout with a blooming artifact - hemorrhagic focus/speck of calcification. Axial T1fs post-contrast (G), the lesion shows heterogeneous enhancement with interspersed non-enhancing necrotic areas within. Coronal STIR (H) shows that the lesion is extending superiorly to the epigastric region. ADC: apparent diffusion coefficient; T2WI: T2 weighted image; STIR: short tau inversion recovery; DWI: diffusion-weighted imaging; GRE: gradient echo sequences; T1FS: T1 weighted fat-saturated image; O-RADS: Ovarian-Adnexal Reporting and Data System
